# Tottenham Hospital: Bazaar for the Ladies' Association

**Published:** 1902-11-01

**Authors:** 


### The Institutional Workshop.

### TOTTENHAM HOSPITAL: BAZAAR FOR THE LADIES' ASSOCIATION.

A two days' bazaar, organised by the Ladies' Association of Tottenham Hospital, was held on Wednesday and Thursday, last week, in aid of the Maintenance and Samaritan Funds. The bazaar took place in Bruce Castle, a fine old country house with a history that goes back to Bruce himself, and is interesting as having a room in which Sir Rowland Hill is said to have elaborated his scheme for Penny Postage. The house, which, with its surrounding park, is now held in trust by the Urban District Council for the people of Tottenham, lent itself admirably to the purpose. Stalls were contributed from Cheshunt, Waltham Abbey, Edmonton, Tottenham, St. Anne's, etc., and the small octagonal room, sacred to Penny Postage fame, was labelled "Post Office." The services of the Blue Hungarian Band were secured by a lady as her contribution to the funds, and during the evening concerts and limelight pictures (including some hospital scenes) were among the various attractions.

The Sale was opened by Sir Henry Burdett, K C.B., who was accompanied by Lady Burdett.

The Rev. E. A. B. Sanders, in introducing Sir Henry, said that in doing so he was introducing no novice in hospital work, indeed he supposed there were few greater authorities than Sir Henry, to whom he looked for sound practical advice of permanent use to those responsible for the conduct of the hospital. As Vicar of an adjoining parish which in a very short term of years had grown from a village into a place of over 50,000 inhabitants, he could not only speak with authority as to the need for the Tottenham Hospital, but he could testify from personal knowledge to the benefits received there by many of his own parishioners. He could not speak too highly of the Ladies' Association, and he was glad to know that it was in a flourishing condition.

### A Threefold Increase of Receipts.

Sir Henry Burdett said that it gave him very great pleasure to be in Tottenham again after an absence of about three years. It would be in the remembrance of some present that in January, 1899, when the Charity Commissioners had arranged a scheme with reference to the hospital, there was much diversity of opinion in the district, and serious difficulty as to starting the hospital under the new auspices. He therefore thought it would be interesting and also desirable (particularly as nothing could be done in this life without encouragement to go forward) if he were to remind his hearers of what was their financial position in 1899, and compare it with what it was to-day. Three years ago the income of the hospital was £1,380, to-day it was £4,430, so that they had increased more than threefold. The expenditure was £5,000, leaving a deficiency of nearly £3,800; to-day it was only £4,146, so that income and expenditure, on the basis of the report for 1901, from which he took the figures, were on a satisfactory financial foundation. He wished, further, to point out that although the hospital showed a balance on the right side, it had expanded its work. Three years ago there were 24 beds, to-day between seventy and eighty; and if it were asked how and why this extra work had been accomplished on an expenditure which was less than that shown when there were only 24 beds, he would remind his hearers that whereas the average cost was £5 4s. a year in 1899, it had been already reduced to £4 15s. per patient. The further reduction was so great that the inquiry was at once made, "What does it mean?" It meant that the alteration which changed the institution into a hospital pure and simple was a wise and proper one; it gave those who were responsible an opportunity of making the hospital a really good one, while they had at the same time extended its usefulness to the poor by making the money go twice as far as it formerly did.

### A Million and a Half Days.

Owing to the development of medical treatment, there had been added to the working days of the wage-earning classes in London (as represented by the saving of time they were now resident in hospital) one and a half million days. This was a significant fact. How did Tottenham stand in respect to the matter? The average length of stay last year was 24 days; this year it was 23; and from facts which he had taken from last year's report there was no doubt the average would be brought down to something like that of the big London hospitals, namely, 19 days.

### Population Imported Wholesale.

He would like to say how much he regretted the absence of the chairman of the hospital, Mr. Cory-Wright, because a bazaar was not only a means of getting money, but an opportunity for the meeting together of all classes. Just a word or two about the needs of the district: it had a population of 300,000, and on a very modest computation there should be a hospital of something like 300 beds. How were the people of Tottenham going to provide these beds? He observed with interest that although the population was already so large, it was about to be increased through the action of the London County Council, which proposed to give Tottenham a large additional population—some 40,000—from the congested London districts. Now there was no one who, from a knowledge of hospitals not only in this country but in all parts of the world, was more confident than he himself was, that the voluntary system was the only one that should prevail in a Christian country; but in view of this wholesale importation of population he felt that a just demand might be made on the London County Council not only to provide dwellings for the healthy, but also hospital buildings for the accommodation of these people in sickness. He hoped the committee of the hospital would agree with this point of view; he certainly thought that as the majority of the members of the London County Council plumed themselves upon their progressiveness, there was reason to hope they might at any rate see the force of the argument. He hoped they would not rest content until the required accommodation was provided on the hospital site in new buildings paid for by the County Council.

### A System of Street Captains.

There was another side to the question: What might the district itself do? In a district like Tottenham it was of no use to hope that the hospital would be adequately supported on the old-fashioned lines of trying to get guineas from those who could afford to give them. If success was to be assured, the interest of the poor people must be secured; public opinion must be there to back up the collectors. If the hospital was well conducted (and the gentleman who had already spoken was able to testify to its usefulness) there should be no difficulty about securing public opinion. Everyone was appalled when asked to approach thousands and tens of thousands of persons with a view to getting money, especially when they realised that some of those thousands were, perhaps not unnaturally, suspicious when asked to give money to charity by a stranger.

"You must create confidence in the collectors," the speaker went on; "if you have that, I make bold to say, with my experience of Highbury and other districts, I think you will get all the money you may need." London, said Sir Henry, wanted more neighbourliness. In his visits to sick people he had always been touched at finding how sickness brought out this quality. There was nothing like the whole-hearted generosity of one poor man to another.

Now how was this confidence to be gained? He would suggest that all streets should be marked out and assigned to captains, one for each street. Whether the captain should be a lady or a gentleman was a matter for the particular district to decide; it might be well to select the person in each street who was most trusted. This captain would be asked to give a few hours to visiting every house in the street once a year, to see every person, and to ask them, on the Scotch system, to write their names in a book and say how much they would give. He believed that under the organisation of Mr. Cory-Wright and the secretary it would be found that this scheme would be a most successful one. He was not asking for an impossibility, for, assuming that there were 200 beds in the hospital, each bed requiring some £60 to £70, the sum to be raised would be £14,000. Towards this they could at present show £4,000 a year. Money would also be wanted for building, but some of this ought to come from the London County Council.

### The Hospitals Must Come to the People.

Confucius used to maintain that if you were good and strong and pure enough you would attract all men to yourself; that was what the Tottenham Hospital ought to be. If they could say "We have the income, and we only want the building for the suffering poor," he believed there would be no difficulty. "Surely," said the speaker, "what Highbury did you can do." In the case of Highbury, he continued, the leaders of the medical profession had greatly questioned the wisdom of moving a hospital out of London; they had indeed said to him, "What are you doing, Burdett? It is dangerous to medical prestige to take a hospital out of London." He replied, "You have no right to compel the poor, who have so many claims upon their slender resources, to travel long distances, the bus-fares making a strain upon their limited resources; we must have a better distribution of hospital accommodation." In this connection it was most satisfactory to know that in five years they not only built a hospital at Highbury costing £100,000, but had put aside £30,000 as a reserve fund. He had always felt that if encouragement were wanted, that instance supplied it. The people of Tottenham and the neighbourhood had already done much, they had gone some distance on the road they had mapped out for themselves. The work they had undertaken was, he knew, regarded with some criticism in the district three years ago. But they had now secured unity, they all believed now that the hospital was a good and necessary one, and they wanted it to succeed. It was at length their own; they could manage their own affairs, and they had an excellent medical staff, consisting of highly qualified gentlemen whom they knew and esteemed.

### Hints for the Ladies' Association.

There was one other point. There was a great deal of extravagance in charity to-day, but here there was none, because it was the literal truth that the money was actually needed—200 beds were actually required by the poor. The audience, he was glad to see, consisted mainly of ladies, and he could quite understand that their thoughts might be thus summed up: "Of course, this gentleman comes down and opens our bazaar, and talks about the men, and gives a great deal of time to their work; but where do we come in?" Now he hoped they would believe that he wanted to encourage the women. The women had done their part too. What was this bazaar for? It was to obtain contributions in aid of the Maintenance Fund and the Ladies' Samaritan Fund. He knew how much the success of Highbury was due to the Ladies' Association, especially to the extraordinary zeal of one lady, Mrs. Harkness, and he believed that if the ladies would devote their attention to the Samaritan side of the hospital's work, they would find great scope for usefulness. They should endeavour to get patients out of the hospital and into the country as soon as possible; the worker who had lost a limb should be provided with surgical appliances without delay; the poor man whose clothes had been injured should be supplied with a new suit in order that he might seek employment as soon as he was cured; and to the very poor, out of knowledge of the circumstances and kindness of heart, temporary aid might be given, and would do an enormous amount of good. He looked to the Ladies' Association for that touch of human sympathy which would kindle the bright light of hope in many of the sick with whom they spent their leisure. After all, the view that must be driven home to the wealthy was a selfish view, and that was that their comforts in sickness, and even the ability when in health to get all the amusement out of life that they wanted, and even the power to go on living in leisurely idleness, would be impossible were it not for the hospitals. It was in the hospitals that medical skill and nursing were brought to their highest development, and he would say to the rich, "Have a care that you give something like your fair share of support to the hospitals, or you may find yourself in straits when you are sick." But there was a higher view, and that was that every person who had been blessed by God with brains or any kind of thinking apparatus worthy of the name, should regard it as a duty and privilege to give some service on behalf of the wayfaring man who, through no fault of his own, was stricken down in God's providence with some terrible disease.

### The Key-note.

Sir Henry concluded by saying that he hoped the work would be a success from a financial point of view, but he hoped still more that by means of the Ladies' Association and the system of street captains which he had outlined and with the sympathy and confidence of the whole population, the work would extend and go forward, so that it might become an object-lesson to the whole of London and the British Empire. It was said that it took a soul to make a body; and he hoped that he might leave behind him a feeling that what he had said, of everybody doing a little to alleviate the sickness and suffering around them, would form the keynote of their future endeavour. As they had increased their resources three-fold in three years, what might they not do in five? At the end of that time they would perhaps ask him to come again, and he was confident that he would find such work had been accomplished as would be an example to all hospitals.

Sir Henry then declared the bazaar open, and expressed his intention of visiting each of the stalls in turn, and making the personal acquaintance of the stallholders.

The Rev. F. B. Johnston said he was sure that he expressed the feelings of the audience when he stated that they were all extremely grateful to Sir Henry and Lady Burdett for the sympathy they had shown by their presence there. He himself believed that if the hospital were adequately supported it might be brought up to the standard of, and the work compare favourably with that of, any London hospital. It was sorely needed in the centre of a large and increasing population 300 beds would be by no means too many. Urgent cases had now to be refused, which could be dealt with if sufficient accommodation were provided.

Dr. May said that Sir Henry Burdett gave the committee a great deal of help three years ago, and he felt sure that his words would animate the people of Tottenham and the district to fresh exertions on behalf of the hospital.

The presentation of a basket of flowers to Lady Burdett completed the opening ceremony, and business began to move briskly at the stalls.

